# T155. SEPARABLE AND REPLICABLE NEURAL STRATEGIES DURING SOCIAL BRAIN FUNCTION IN PEOPLE WITH AND WITHOUT SEVERE MENTAL ILLNESS

**DOI:** 10.1093/schbul/sby016.431

**Published:** 2018-04-01

**Authors:** Colin Hawco, Robert Buchanan, Navona Calrco, Benoit Mulsant, Joseph Viviano, Erin Dickie, Miklos Argyelan, James Gold, Marco Iacoboni, Pamela DeRosse, George Foussias, Anil Malhotra, Aristotle Voineskos

**Affiliations:** 1 University of Toronto; 2 Maryland Psychiatric Research Center; 3 The Zucker Hillside Hospital; 4 David Geffen School of Medicine at UCLA

## Abstract

**Background:**

The case-control design and disease heterogeneity may be major limiting factors impeding biomarker discovery in brain disorders, including serious mental illness such as schizophrenia spectrum disorder (SSD) or bipolar disorder (BPD). We propose that this heterogeneity represents an opportunity for discovery by uncovering relevant biologically driven sub-types within disorders. Individuals with schizophrenia spectrum disorder (SSD) have deficits in social cognition related to poor functional outcome.

**Methods:**

A total of 109 SSD and 70 matched healthy controls (HC) were recruited across three sites. Participants performed an fMRI task in which they observed or imitated emotional faces. For each participant, an individual pattern of activity (Imitate > Observe for emotional faces) was identified. Hierarchical clustering (Ward’s method) identified clusters of individuals with similar patterns of activity. We then examined whether new data-driven groups of participants (based on patterns of brain activity) demonstrated performance differences on a batter of social and neuro cognitive tests completed out of the scanner. As a validation of the importance of cluster membership, Euclidean distance was compared between participants to members of their own cluster, diagnosis, or site. The clustering analysis was repeated on a replication sample consisting of 32 SSD, 37 euthymic BPD, and 39 HC.

**Results:**

Three clusters with distinct patterns of neural activity were found. Cluster one (24 HC and 44 SSD) represented ‘typical activators’ (lateral frontal and parietal activity). Cluster two (21 HC and 31 SSD) were identified as ‘hyper-activators’, showing more intense and extended activity. This was interpreted as a ‘compensatory’ response of over-activation related to impaired neural circuits, such as is seen in aging. Interestingly, cluster three (25 Controls and 35 SSD) showed a very atypical pattern, including suppression of activity during imitation in regions involved in the default mode network and/or higher order social cognition (e.g. theory of mind). This group also had improved social cognitive performance relative to the other clusters. Participants were found to have more similar patterns of brain activity to members of their cluster rather than to members of their diagnostic group or scanning site. Importantly, when clustering was applied to the replication sample, the same three patterns (typical activators, hyper activators, and deactivators) were identified.

**Discussion:**

In independently collected samples, our findings demonstrate different patterns of neural activity among individuals during a socio-emotional task that were independent of DSM-diagnosis or scan site. Our findings may provide objective neuroimaging endpoints (or biomarkers) for subgroups of individuals in target engagement research aimed at enhancing cognitive performance independent of diagnostic category.

